# Noise and crosstalk in two quorum-sensing inputs of *Vibrio fischeri*

**DOI:** 10.1186/1752-0509-5-153

**Published:** 2011-09-29

**Authors:** Pablo D Pérez, Joel T Weiss, Stephen J Hagen

**Affiliations:** 1 Department of Physics, University of Florida, Gainesville FL 32611-8440, USA

## Abstract

**Background:**

One of the puzzles in bacterial quorum sensing is understanding how an organism integrates the information gained from multiple input signals. The marine bacterium *Vibrio fischeri *regulates its bioluminescence through a quorum sensing mechanism that receives input from three pheromone signals, including two acyl homoserine lactone (HSL) signals. While the role of the 3-oxo-C6 homoserine lactone (3OC6HSL) signal in activating the *lux *genes has been extensively studied and modeled, the role of the C8 homoserine lactone (C8HSL) is less obvious, as it can either activate luminescence or block its activation. It remains unclear how crosstalk between C8HSL and 3OC6HSL affects the information that the bacterium obtains through quorum sensing.

**Results:**

We have used microfluidic methods to measure the response of individual *V.fischeri *cells to combinations of C8HSL and 3OC6HSL. By measuring the fluorescence of individual *V.fischeri *cells containing a chromosomal *gfp*-reporter for the *lux *genes, we study how combinations of exogenous HSLs affect both the population average and the cell-to-cell variability of *lux *activation levels. At the level of a population average, the crosstalk between the C8HSL and 3OC6HSL inputs is well-described by a competitive inhibition model. At the level of individual cells, the heterogeneity in the *lux *response depends only on the average degree of activation, so that the noise in the output is not reduced by the presence of the second HSL signal. Overall we find that the mutual information between the signal inputs and the *lux *output is less than one bit. A nonlinear correlation between fluorescence and bioluminescence outputs from *lux *leads to different noise properties for these reporters.

**Conclusions:**

The *lux *genes in *V.fischeri *do not appear to distinguish between the two HSL inputs, and even with two signal inputs the regulation of *lux *is extremely noisy. Hence the role of crosstalk from the C8HSL input may not be to improve sensing precision, but rather to suppress the sensitivity of the switch for as long as possible during colony growth.

## Background

Quorum sensing is a mechanism of bacterial gene regulation that is based on the release and detection of diffusible chemical signals. It is classically described as a population-sensing scheme: the bacteria release a pheromone (autoinducer) into their environment, and the accumulation of this autoinducer is an indicator of a high population density, triggering changes in phenotype. However it has become increasingly apparent that bacterial quorum sensing (QS) behaviors are often more complex than simple population-counting [1-4]. Many QS regulatory networks employ multiple receptors that receive signals from different autoinducers, forming interacting detectors that may act sequentially or in parallel to regulate downstream genes. The design principles of these multi-input systems remain mysterious: One of the interesting puzzles in the study of QS is to understand what benefit or information an organism can gain from combining multiple autoinducer inputs, and how information from different inputs is processed to generate a useful output [5,6].

Here we investigate this question for two autoinducer inputs in *Vibrio fischeri*, a γ-proteobacterium that uses QS to regulate bioluminescence as well as other behaviors that are important to colonization of its symbiotic host animal. These two autoinducers exhibit a competitive or antagonistic interaction in regulating the *lux *operon that controls bioluminescence. We use microfluidic and single-cell methods to observe how combinations of the two autoinducer signals affect the bulk or average output of the QS network, as well as the cell-to-cell variability in the activation of *lux*. We ask whether different combinations of signal inputs that produce the same average response across a population also produce the same response from individual *V.fischeri*, and therefore whether the *lux *system gains additional information from the presence of an additional signal.

**Figure 1:**
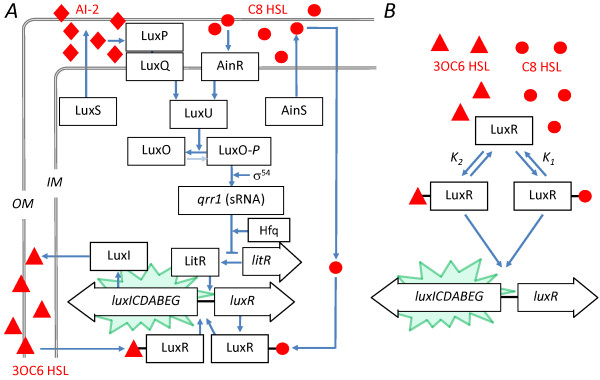
**Schematic of QS regulation of *V.fischeri *bioluminescence and competitive model**. (*A*) QS regulation of bioluminescence in *V.fischeri *uses three autoinducer channels [8]. The autoinducer 3OC6HSL is synthesized by LuxI and binds to LuxR to form a transcriptional activator for the bioluminescence genes *luxCDABEG*. Two more autoinducers (C8HSL and AI2) drive the phosphorelay that regulates production of LuxR as well as other colonization behaviors. (*B*) A simplified model considers only competitive interaction between 3OC6HSL and C8HSL, as proposed by Kuo *et al*. [13] and Lupp *et al*. [11]. The receptor LuxR binds the autoinducers C8HSL and 3OC6HSL to form multimeric complexes (of degree *m *and *n *respectively) which activate *lux *transcription. In our fit we omit *lux *activation by the C8HSL complex of LuxR.

Bioluminescence in *Vibrio fischeri *is generated by the *lux *operon *luxICDABEG*, which encodes the bacterial luciferase as well as enzymes for production of the luciferase substrate [7]. It is regulated by three QS channels [8] (Figure 1). Most well known is the LuxI/R mechanism. LuxI is the synthase of the autoinducer *N*-3-oxohexanoyl-*L-*homoserine lactone (3OC6HSL), which interacts with its cognate receptor LuxR to form a transcriptional activator for the *lux *operon. Threshhold concentrations (nM) of 3OC6HSL induce *V.fischeri *bioluminescence.

The second QS system in *V.fischeri *is the AinS/R system (Figure 1). It uses the synthase AinS to produce the signal *N*-octanoyl-*L*-homoserine lactone (C8HSL). C8HSL interacts with its cognate receptor kinase AinR to initiate a phosphorelay signaling cascade (involving LuxU, LuxO, and a small RNA) that interrupts negative regulation of LitR, an activator of LuxR. In addition to regulating luminescence via LuxR, AinS/R also regulates a number of other behaviors, such as motility and acetate utilization [9], that are important to successful colonization of the symbiotic host [10,11].

The third QS system in *V.fischeri *differs from the first and second because it does not employ an acyl homoserine lactone (HSL) autoinducer. Instead the autoinducer is a furanosyl borate diester (AI2) that is synthesized by LuxS and detected by LuxP and LuQ. The signal feeds into the same phosphorelay channel that detects the C8HSL autoinducer of the AinS/R system. AI2 influences luminescence (and presumably also noise in luminescence) via its downstream effect on LuxR expression. However the AI2 input makes a relatively small contribution to luminescence regulation and colonization [12], especially in comparison to the HSL autoinducers C8HSL and 3OC6HSL. Mutants deficient in production of the HSL autoinducers produce only very low, basal luminescence if any [13,14]. Therefore, although AI2 may be important in interspecies communication [2], we have not included it in this study.

Interestingly, the second autoinducer, C8HSL, also acts on luminescence through an additional route, where it bypasses the phosphorelay and interacts directly with LuxR to activate *lux *expression (Figure 1). Therefore, while QS control of *V.fischeri *luminescence primarily occurs through the C8HSL and 3OC6HSL autoinducers, the effect of the two HSLs is not simply additive. The two routes of C8HSL action lead to a complex crosstalk between the AinS/R and LuxI/R systems. Generally, in a *V.fischeri *culture lacking 3OC6HSL, the addition of C8HSL induces bioluminescence. By contrast, in the presence of 3OC6HSL, the addition of C8HSL suppresses bioluminescence. Furthermore C8HSL appears to influence luminescence largely through direct interaction with the *lux *operon, rather than through the phosphorelay channel [13,15]. These findings suggested a competitive inhibition model [11,13] in which C8HSL modulates the bioluminescence by competing for the 3OC6HSL receptor LuxR (Figure 1): both C8HSL and 3OC6HSL are capable of binding to the receptor LuxR and activating transcription of the *lux *genes [16], but the C8HSL-LuxR complex is a less effective activator than the 3OC6HSL-LuxR complex.

The sensitivity of LuxR (as an activator of the *lux *genes) to C8HSL *vs*. 3OC6HSL is readily tunable through single-residue mutations [17], indicating that crosstalk could be minimized if it impaired optimal regulation of bioluminescence. In fact, interaction between AinS/R and LuxI/R not only exists but is strain-dependent, as the luminescence of *V.fischeri *mutants lacking the C8HSL synthase (*ainS *mutants) behaves differently for strains derived from different symbiotic host animals. While the *ainS *mutation suppressed the luminescence of a strain extracted from the squid host *Euprymna scolopes *[11], the *ainS *mutation accelerated the induction of luminescence in a strain gathered from the fish host *Monocentris japonicus *[13]. Furthermore, genomic analysis of *V.fischeri *strains derived from squid *versus *fish hosts showed that while most genes are highly conserved, luminescence activation and the regulatory targets of the LuxI/LuxR system exhibit significant divergence between strains [18,19].

The strength of the crosstalk between C8HSL and 3OC6HSL, and the tuning of this interaction in strains that occupy different symbiotic environments, suggests that it is not incidental but rather that it provides an adaptive benefit. We should ask how a system of two HSLs, working in opposition to each other, improves the regulation of bioluminescence. For example, Kuo *et al*. noted that synthesis of C8HSL will delay the induction of luminescence early in growth, conserving the energy resources of the organism [13]. Lupp and Ruby [10], noting that C8HSL regulates colonization factors in addition to luminescence, suggested that AinS/R and LuxI/R act sequentially so that the maximum induction of luminescence occurs after host colonization is initiated. It is still puzzling however that AinS/R should exhibit such strong crosstalk with LuxI/R in regulating bioluminescence, since the same average delay in luminescence could presumably be achieved through a higher activation threshold for 3OC6HSL.

Here we have investigated how combinations of C8HSL and 3OC6HSL signals affect the response of the *lux *operon at the individual cell level. Our recent study of the bioluminescent emission from individual *V.fischeri *found that the response of individual cells to defined concentrations of exogenous 3OC6HSL (alone) was extremely heterogeneous in the overall magnitude of luminescent emission and in the time scale for response to the HSL signal [20]. Although the LuxI/R system exerts good control of the average bioluminescence of a *V.fischeri *population, it provides only weak control of individual cell behavior. Therefore we ask whether the presence of two signals, C8HSL and 3OC6HSL, provides an additional dimension of control at the individual cell level, *i.e*. whether combinations of HSLs elicit a less noisy and more precise response in individual cells and therefore whether the second HSL signal improves the sensing precision of the QS system.

Although we measured individual cell bioluminescence directly in our previous study, here we use a fluorescent reporting strain (JB10) of *V.fischeri*, containing a chromosomal *gfp *reporter of *lux *operon activation. We first show that the activation of JB10 *GFP *fluorescence in the presence of C8HSL and 3OC6HSL is described quantitatively by the competitive inhibition model of Figure 1. The empirical parameters from the model define the average response and provide the basis for microfluidic single cell studies, in which we apply combinations of C8HSL and 3OC6HSL autoinducers and measure the cell-to-cell variation in the *lux *response. From the observed distributions we can determine whether combinations of HSL inputs improve the precision of individual cell response. Combining the single cell observations with the parametrized model we can also estimate the throughput of information from the HSL signal inputs to the overall *lux *output.

## Results

### Modeling the lux response

**Figure 2:**
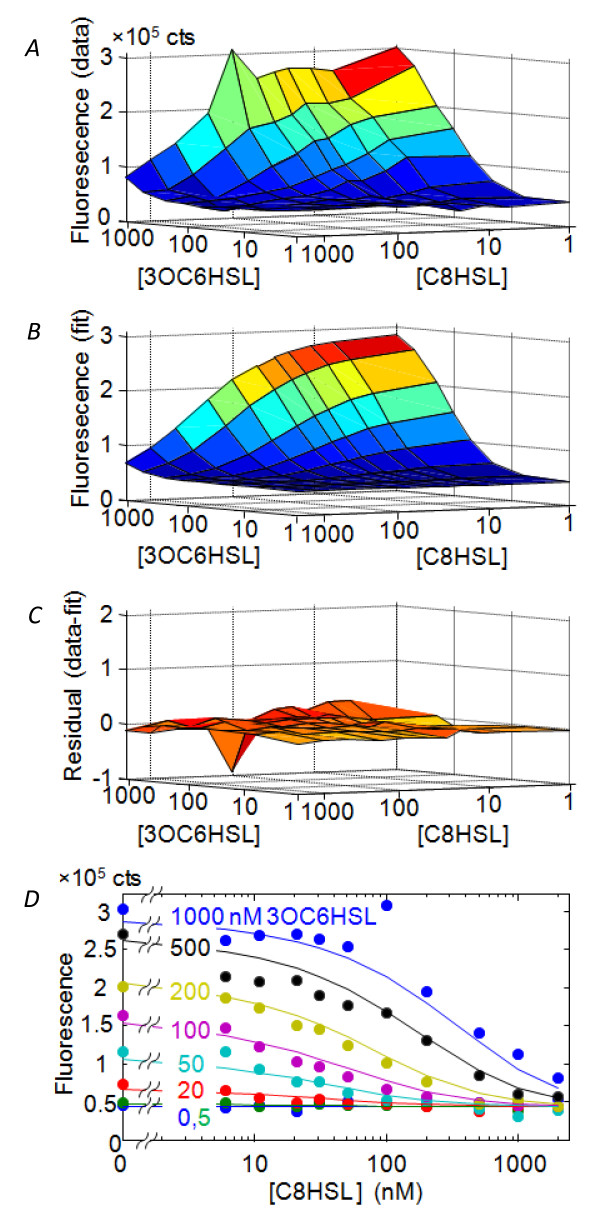
**Data and fit for *lux *activation of JB10 strain by two autoinducers**. The figure shows the population-average (bulk measurement) response of combinations of exogenous C8HSL and 3OC6HSL autoinducers, as measured by GFP fluorescence of strain JB10. (*A*) Data at OD = 0.15 cm^-1^, (*B*) fit to competitive inhibition model, (*C*) residual from fit, and (*D*) Data and fit sectioned at constant [3OC6HSL].

**Figure 3:**
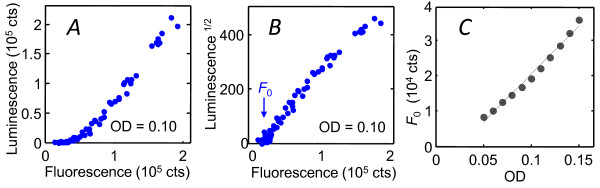
**Correlation between luminescence and GFP fluorescence of JB10 strain**. (*A*) Correlation between bioluminescence *L *and GFP fluorescence *F *of the JB10 strain, as observed in a bulk (well-plate) experiment. Data were collected at *OD *= 0.10 in the presence of exogenous C8HSL and 3OC6HSL spanning a range of concentrations from zero to ~ 1000 nM. (*B*) Same data plotted as *L*^1/2 ^vs *F*, showing the approximate power law of Eqn. (1). (*C*) The HSL-independent intercept *F_0 _*in Eqn. (1) grows in proportion to optical density during early growth.

Here our goal is to construct a mathematical representation of the interaction between HSL signals by fitting bulk (well-plate) data to the competitive-binding model shown in Figure 1 (see *Methods*). The HSL-induced bioluminescence of *V.fischeri *strain JB10 observed in the well-plate assay is very similar to that observed in wild type strains [11,13,15]: C8HSL weakly activates the bioluminescence in the absence of 3OC6HSL, while it represses bioluminescence in the presence of 3OC6HSL. 3OC6HSL consistently activates bioluminescence. Figure 2 shows the GFP fluorescence of JB10 as a function of HSL inputs. Since the *gfp *reporter is inserted into the *lux *operon of JB10, we expect the GFP fluorescence to correlate closely with bioluminescence over the full range of C8HSL and 3OC6HSL inputs. In principle we then have the choice of fitting the model to either the luminescence *L *or the fluorescence *F*, as fluorescence and luminescence reporters are both regarded as reliable measures of gene expression [21]. However Figure 3 shows that the correlation between the fluorescence and luminescence of JB10 is strong but it is not linear: at *t *= 10 hrs, when the bioluminescence response spans a dynamic range of ~3-4 decades, the fluorescence spans only ~ 20-fold. In fact, the empirical relationship between fluorescence *F*([3OC6HSL], [C8HSL]) and luminescence *L*([3OC6HSL], [C8HSL]) is nearer to a power law

(1) $$L^{1∕2}\approx \alpha \left(F-F_{0}\right).$$

Here *a *is a proportionality constant and *F_0 _*describes a baseline fluorescence that is present even at the lowest activation levels where the luminescence is undetectable (*L *≈ 0). As the baseline *F*_0 _grows with the population density (Figure 3) we interpret it as either a baseline expression of *lux *- occurring independent of HSL activation [14] - or autofluorescence of the cells.

The power law in Eqn. (1) suggests that the luminescence intensity is affected by the association equilibrium of LuxA and LuxB, which form the bacterial luciferase heterodimer [7]: In a simple dimer association model, the concentration of enzymatically active luciferase (∝*L*) should scale as the product of the LuxA and LuxB concentrations. We also expect that *luxA*, *luxB*, and *gfp *should all be expressed at similar levels as they are all under the control of the same promoter. Therefore *L *should correlate with the square of the GFP concentration (∝*F-F*_0_), leading to Eqn. (1). From this perspective fluorescence *F *(relative to its background *F_0_*) is preferable to *L *as a reporter of *lux *activation and should give a better fit to a physical model. Therefore we based our analysis on the fluorescence *F *data. However the model also fits *L*^1/2 ^with virtually the same parameters as it fits *F *(Table 1).

**Table 1:** Parameter values for the competitive inhibition model

Fit	k_1 _(nM)	*m*	k_2 _(nM)	*n*
1	39 ± 8	1.1 ± 0.4	163 ± 15	1.35 ± 0.05

2	46 ± 2	1.2 ± 0.2	179 ± 6	(= *m*)

3	38 ± 5	0.8 ± 0.2	118 ± 11	1.6 ± 0.06

Parameter values for the competitive model were obtained by fitting Eqn. (6) to the GFP expression data (Figure 2), for a bulk culture of *Vibrio fischeri *strain JB10 at density OD = 0.05-0.15 cm^-1^. The first row contains parameters obtained by fitting the GFP fluorescence data (*F*) with the four-parameter model described in the text. The second row contains parameters obtained by fitting the fluorescence data under the constraint that *m *= *n*. The third row contains fit parameters obtained when the four-parameter model is fit to the luminescence data (actually *L*^1/2^) rather than to the fluorescence *F*. (See Eqn. (1)).

Fitting the competitive inhibition model to GFP fluorescence data at a range of optical densities early in growth (OD = 0.05-0.15 cm^-1^) gives a very satisfactory fit with minimal spread in the parameter values (Figure 2 and Table 1). Averaging the fit parameters obtained for *F *data over the OD range produces the surface *F*([3OC6HSL],[C8HSL]) shown in Figure 2. Using this model surface we can predict the (average) *lux *response under any HSL condition and identify HSL combinations of interest for single-cell studies.

### Microfluidic studies of individual cells

**Figure 4:**
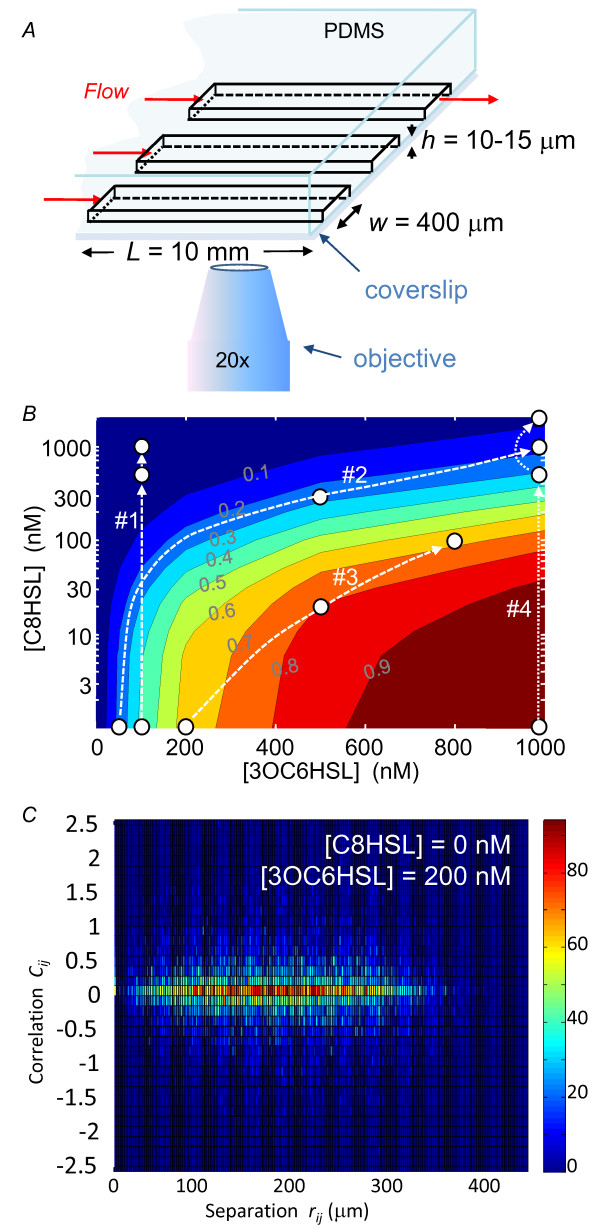
**Microfluidic measurements of GFP fluorescence**. (*A*) Schematic of microfluidic device for measuring *lux *expression in individual *V.fischeri*. Growth medium containing exogenous HSL flows through three parallel rectangular channels that are cast into the lower surface of a PDMS block. Live cells in channels adhere to the glass window (coverslip) that seals the channels from beneath, and are observed in an inverted microscope. The shallow ratio of channel height to width (*h/w *~ 0.02-0.04) ensures a uniform flow velocity profile across the width and length of each channel. (*B*) Contour map of *lux *activation *F *versus HSL input. The white circles show HSL combinations applied to the cells during the microfluidic experiments described in the text. The contour labels show the activation fraction above the base level, *i.e*. (*F-F*_0_)/max(*F-F*_0_), as derived from the bulk measurements and competitive inhibition model. (*C*) Histogram comparing the correlation *C_ij _*(Eqn. (7)) in *gfp *expression of a pair of cells (*i, j*) to the physical separation *r_ij _*= √(*x_ij_*^2^+*y_ij_*^2^) between those cells. The color of each bin indicates the number of cell pairs (*i, j*) whose physical separation and brightness correlation fall within that bin. Pairs of near-neighbor cells are not more correlated in their *lux *activation than pairs of distant cells.

By loading *V.fischeri *cells into a three-channel microfluidic device (Figure 4) on a fluorescence microscope we can observe simultaneously three groups of cells subject to different combinations of HSL inputs and characterize the heterogeneity of their GFP response. The contour map of Figure 4 shows the HSL combinations used in four such experiments. Two experiments explore the heterogeneity along contours of near-constant *lux *activation while two experiments use signal combinations that cross contour lines.

**Figure 5:**
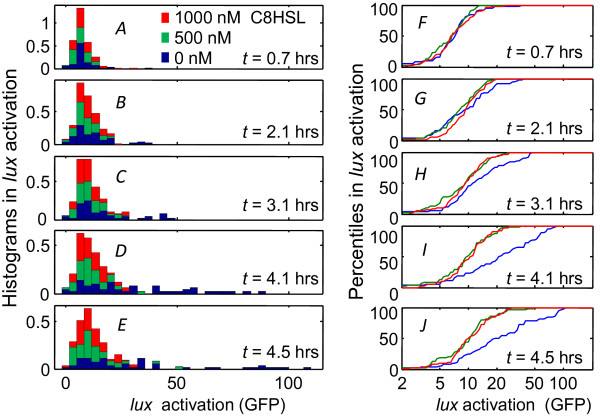
**Temporal response of the *lux *activation histogram**. (*A*)-(*E*) Progression of the GFP expression histogram for individual cells in the microfluidic chamber, as measured for the three signal combinations indicated as Experiment #1 (*i.e*. 100 nM 3OC6HSL and variable C8HSL) in Figure 4B; (*F*)-(*J*) Percentiles in *gfp *fluorescence of individual cells under different HSL conditions. Each curve shows the cumulative area under the corresponding histogram in panels (*A*)-(*E*) (and with the same color scheme). In the absence of C8HSL the distribution broadens (and increases in mean value) over 3-4 hrs, while the presence of C8HSL suppresses this response.

Experiment #1 applied three different C8HSL concentrations - along with 100 nM 3OC6HSL - to the three device channels at time *t *= 0. Initially all cells exhibit a weak fluorescence with a narrow distribution. In the absence of C8HSL, the response evolves over ~3-5 hrs to give a broad and distinctly non-Gaussian distribution that extends over an order of magnitude in GFP fluorescence, with a minority of cells becoming far brighter than the average. By contrast, when C8HSL was present at 500 nM or 1000 nM, the average cell fluorescence increased slightly over time, but the distribution still remained narrower than in the absence of C8HSL. Since the high C8HSL conditions (*i.e*. C8HSL = 1000 nM and 3OC6HSL = 100 nM) induced virtually no *lux *response in our bulk experiments, we interpret the weak response as the baseline autofluorescence *F_0 _*of Eqn. (1). Figure 5 shows that C8HSL does not simply reduce the average fluorescence, but rather reshapes the distribution by suppressing the development of the highly heterogeneous (noisy) activated response.

**Figure 6:**
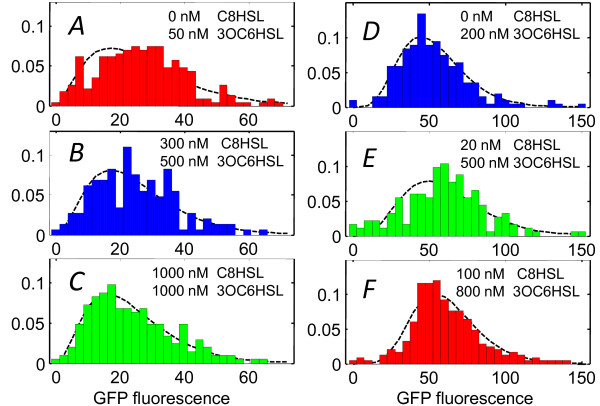
**Cell-to-cell heterogeneity at fixed average activation**. Cell fluorescence brightness distributions measured for experiments #2 and #3 in Figure 4B. (*A*)-(*C*) Distributions collected for two-HSL combinations that generated ~25% of full *lux *activation (Experiment #2) and (*D*)-(*F*) distributions collected for combinations that generated ~60% of full *lux *activation (Experiment #3). The dashed curves show maximum likelihood fits to a gamma distribution. Each histogram is derived from roughly 200 individual cells.

Experiments #2 and #3 examined whether different combinations of C8HSL and 3OC6HSL that induce the same average response also elicit the same degree of heterogeneity. The selected HSL conditions for #2 and #3 (Figure 6) follow two contours in Figure 4 corresponding to roughly 25% and 60% of full activation, respectively. Although the variance σ^2 ^in GFP expression increases relative to the mean μ at higher activation levels, we find that different combinations of HSLs that produce similar overall average fluorescence *F *also produce similar distributions. That is, for a given degree of activation, the distribution of individual cell responses does not appear sensitive to the particular combination of HSL signals that induced that response. We also find that the *lux *response develops on the same time scale in all three channels, regardless of the relative proportions of 3OC6HSL and C8HSL. This is consistent with prior findings that C8HSL signaling through the AinR/S route is not essential for luminescence [22]. C8HSL acts on the same time scale as 3OC6HSL as it primarily regulates luminescence through a direct association with LuxR.

### Mutual information between inputs and output

**Figure 7:**
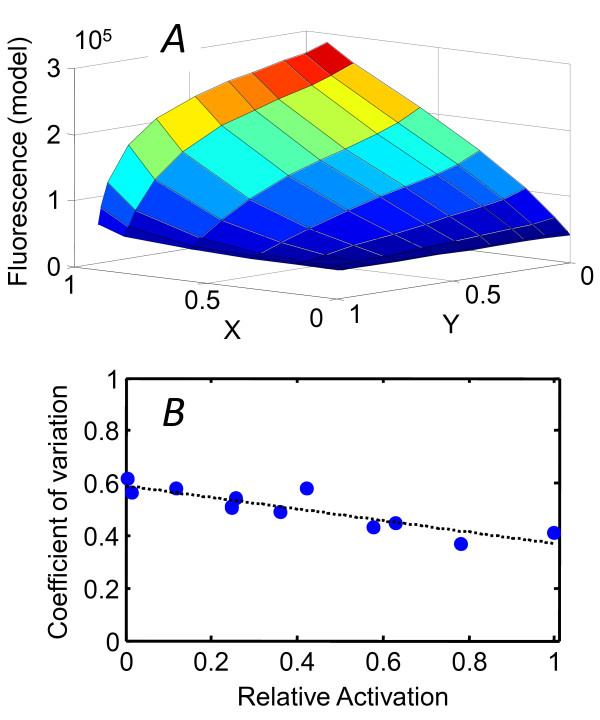
**Response surface *F*(*X, Y*) and the coefficient of variation**. (*A*) The (population-average) response surface *F*(*X,Y*) generated by the competitive model with the change of variable, Eqn. (8). (*B*) The measured coefficient of variation *CV *= σ/μ in *lux *activation of individual cells declines at higher activation levels. Relative activation is defined as (*F-min*(*F*))/(*max*(*F*)*-min*(*F*)). The dashed line shows a linear fit, used to parameterize *CV *in the calculation of the mutual information *I*(*F; *(*X, Y*)).

The cell fluorescence histograms can be expressed as probability distributions *P*(*F *| ([C8HSL], [3OC6HSL])) for the cell fluorescence *F*, given the HSL inputs. The finding that these distributions are a function of the overall *lux *activation *F*([C8 HSL], [3OC6 HSL]), rather than depending on the C8HSL and 3OC6HSL levels separately (Figures 6 and 7), strongly suggests that the additional (C8HSL) input does not induce a response from the *lux *genes of the individual cell that 3OC6HSL alone could not extract. In this sense the *lux *system does not gain additional information by employing two HSL autoinducers. (This would not be true of other regulatory targets of the phosphorelay controlled by AinS/R and LuxS/P/Q, which is not regulated by 3OC6HSL.) We can quantify the information that is gained by calculating the mutual information, which measures the regulatory precision, or the number of practically distinguishable input/output states, of this regulatory system. One may think of mutual information as measuring the amount by which the uncertainty in the (GFP) output is reduced by knowledge of the HSL inputs (and vice versa) [23-25]. Mutual information has been illuminating in recent studies of information throughput in other single-cell chemical sensing systems such as the multiple-autoinducer QS scheme of *V.harveyi *[25] and the chemotaxis of *Dictyostelium discoideum *[26].

Calculating the mutual information between the cell's environment, as defined by the HSL inputs, and the *lux *output requires a mathematical model for *P*(*F*). For higher activation levels we found that *P*(*F*) is too broad to be satisfactorily represented as a Gaussian, although it is reasonably well-described by a gamma distribution [27]:

(2) $$p\left(F\right)dF=dF\;F^{\left(v-1\right)}\text{exp}\left(-F∕b\right)∕\Gamma \left(v\right)b^{v}$$

The gamma distribution depends on two independent parameters, *ν *and *b*, that depend on the variance σ^2 ^and mean μ of the distribution. The dimensionless parameter *ν *= μ^2^/σ^2 ^completely determines the shape of *P*(*F*). The Fano factor *b *= σ^2^/μ does not affect the shape of the distribution, but it scales the horizontal axis and normalization according to the units of measurement of *F *(*e.g*. protein copy number, GFP fluorescence counts, etc.). Eqn. (2) is an appealing model for *P*(*F*) because it arises naturally in an intrinsic noise model, where mRNAs from one gene are synthesized in a Poisson process, with each mRNA leading to a burst of protein expression [27,28]. Therefore we modeled the observed heterogeneity *P*(*F*) as a gamma distribution that has the same relationship between the ratio σ/μ and the average activation level as we observed in our experiments.

For this calculation we also define two new coordinates, *X *and *Y*, to represent the progress of the two LuxR-HSL binding equilibria:

$$\begin{array}{c} \text{X}={\left[30\text{C}6\right]}^{\text{n}}∕\left({\left[30\text{C}6\right]}^{\text{n}}+{{\text{k}}_{2}}^{\text{n}}\right)\\ \text{Y}={\left[\text{C}8\right]}^{\text{m}}∕\left({\left[\text{C}8\right]}^{\text{m}}+{{\text{k}}_{1}}^{\text{m}}\right) \end{array}$$

(See *Methods*). *X *and *Y *are both confined to the interval 0 → 1. When plotted in terms of these coordinates, the response surface *F*(X,Y) generated by the competitive model has a curved shape (Figure 7) that highlights the asymmetry in the *lux *response to *Y *(*i.e*. to [C8 HSL]) versus *X *([3OC6 HSL]): the sensitivity to *Y *depends on the value of *X*.

The models for *F*(*X, Y*) and *P*(*F*) together provide an accurate mathematical representation of our bulk and single-cell data, from which we can calculate the mutual information *I*(*F*; (*X, Y*)) between the input combination (*X, Y*) and the output *F*. The calculation, described in *Methods*, leads to a very modest *I*(*F*; (*X, Y*)) *≈ *0.53 bits. By contrast, a simple noiseless *ON*/*OFF *switch would transmit precisely one bit. Therefore, while the LuxI/LuxR system provides precise control of the average *lux *response of a population over a range of HSL input concentrations, that response activates so gradually with respect to the input levels, and with such a heterogeneous output, that the individual cell cannot be said to exhibit a clean switching between the *OFF *and *ON *states of its *lux *response.

## Discussion

The functional advantages of multi-input quorum sensing (QS) systems are not generally understood, even though many such architectures are known [5,6]. Because our previous study of *V.fischeri *bioluminescence showed an extremely noisy response to 3OC6HSL alone, we investigated whether the presence of the C8HSL signal in addition to 3OC6HSL affects the noise performance of the LuxI/R system. To accomplish this we first constructed a data-based model for competitive inhibition of 3OC6HSL by C8HSL and used this model to draw a contour map of *lux *activation by the two signals. We then used microfluidic devices to control the chemical environment while we measured the heterogeneity of *lux *response among individual cells. The microfluidic flow chamber allows for extended observation of individual cells as well as precise definition of the exogenous HSL levels, eliminating the possibility of QS circuit autoactivation.

*V.fischeri *luminescence is a model system in quorum regulation, and accordingly it has been the subject of mathematical studies of both deterministic and stochastic QS behavior [29-36]. Most studies have emphasized dynamics and steady states of the *lux *system and 3OC6HSL alone, without considering the role of AinS/R. One exception is the work of Kuttler and Hense [34], who presented a detailed dynamical model for the combined *ain *and *lux *signaling pathway as outlined by Lupp *et al*. [11] and others. The resulting system of ordinary differential equations displays a variety of possible stationary states and dynamics, although these outcomes depend on some poorly-known microscopic parameters that characterize transport, production and degradation of the HSLs, synthase production, and the kinetics of HSL-LuxR complex formation. Interestingly however, those authors found that the experimentally-observed differences in the effect of the *ainS *mutation in squid-derived [11] versus fish-derived [13] strains of *V.fischeri *could arise from relatively minor differences in parameters describing the C8HSL and 3OC6HSL competition for LuxR. Specifically, the relative affinity of the C8HSL-LuxR *vs*. 3OC6HSL-LuxR complex for the *lux *box, and the relative strength of *lux *activation by C8HSL-LuxR *vs*. 3OC6HSL-LuxR, can determine whether an *ainS *mutant will be dark (as in [11]) or show accelerated luminescence response (as in [13]). These parameters influence dynamical effects such as the role of *ainS *in the autoactivation of the LuxI system.

Because autoactivation is not possible in the microfluidic chamber, and because C8HSL primarily affects bioluminescence through its direct interaction with LuxR, we set aside many of these dynamical complexities in modeling our population-averaged data on the effect of different HSL combinations. Instead we used the four-parameter competitive inhibition model (Figure 1) described in *Methods*. The model provides a satisfactory fit to experimental data collected on the fluorescent reporting strain (JB10), although the same model will not describe the bioluminescence as accurately (see Eqn. (1)). From the fit parameters we calculated the fluorescence response surface *F*((C8HSL), (3OC6HSL)), which we used as the basis for microfluidic studies of the heterogeneity in *lux *activation in the presence of multiple autoinducers. We studied the heterogeneity in the *lux *response along contours (of constant fluorescence *F*) or along slices (of varying *F*) in the [C8HSL],[3OC6HSL] plane. In general the heterogeneity varies with the average degree of *lux *expression, with the coefficient of variation (σ/μ) trending downward as activation increases (Figure 7). The variance in individual cell responses appears much less sensitive to the particular combination of C8HSL and 3OC6HSL inputs than to the overall degree of activation. The relative proportions of 3OC6HSL and C8HSL do not appear to influence the time scale for development of the *lux *response. These findings are fully consistent with the competitive inhibition model, if the noise in LuxI/R is controlled by a low copy number of the HSL receptor LuxR [35].

One of the puzzles in our previous study of bioluminescence was that the noise in the 3OC6HSL response was quite large *i.e*. CV = σ/μ≈ 1, especially as a GFP-reporter study found significantly lower noise levels, CV ~ 0.15-0.4 in *V.harveyi *QS [37]. Here it is interesting the empirical Eqn. (1) predicts different noise levels for luminescence *vs*. fluorescence reporters of *lux*. If *F' = F-F_0 _*is the level of fluorescence activation above threshhold, then Eqn. (1) predicts δ*L*/*L *≈ 2 δ*F'*/*F'*, so that the coefficient of variation (CV = σ/μ) should be roughly twice as large for the luminescence as for the fluorescence. Therefore the heterogeneity seen in the single cell fluorescence, which is characterized by CV ≈ 0.4-0.6, is fully consistent with our single cell bioluminescence data. Nevertheless the regulation of *lux *still appears noisier in *V.fischeri *than in *V.harveyi *[37,38].

The antagonistic interaction between HSL signals in *V.fischeri *is an intriguing contrast to the additive signaling found in *V. harveyi *bioluminescence, a model system for QS regulation that lacks the LuxI/LuxR mechanism. In *V.harveyi *three distinct autoinducers are detected by three membrane-bound histidine kinases that feed into the same phosphotransferase LuxU. LuxU controls a phosphorelay cascade that regulates the single output LuxR_VH _(unrelated to *V.fischeri *LuxR). The fact that the circuit merges inputs from both an HSL and a furanosyl borate diester (AI2) autoinducer suggests that it senses both intra-species and interspecies QS signals, possibly functioning as a coincidence detector for the input signals [39]. This could increase the system's resistance to crosstalk from other bacterial QS signals or prevent it from responding in certain habitats. Moreover the autoinducer response is additive and symmetric in the sense that all three receptors contribute positively and in parallel to the output, with two having equal kinase activities, so that the output responds in the same way to activation of each receptor [5,25,37]. Those authors suggested that equal sensitivity to each autoinducer benefits the organism by providing a graded, sequential activation of bioluminescence during growth.

Lupp *et al*. proposed a similar, sequential interpretation [11] for the role of C8HSL and 3OC6HSL in *V.fischeri*. They suggested that C8HSL acts first to stimulate luminescence at intermediate cell densities (as in cultures), activating luxR expression through the AinS/R route and also interacting directly with LuxR. At higher cell densities (as later during colonization) *luxR *remains activated by C8HSL but 3OC6HSL accumulates to sufficient concentrations to interact with LuxR and activate *lux*. It would indeed be remarkable if both *V.fischeri *and *V.harveyi *used multiple autoinducers to achieve sequential activation of *lux*, yet only *V.fischeri *did so by using antagonistic signal inputs.

Alternatively, Kuo *et al*. suggested that the suppressing role of C8HSL served a different function, conserving the energy resources of the organism by delaying the induction of luminescence early in *V.fischeri *growth [13]. One puzzle however is that the same delayed outcome could presumably be achieved by setting a higher threshold for induction by 3OC6HSL, making the second signal unnecessary.

We cannot fully interpret bioluminescence regulation in *V.fischeri *without considering its symbiotic context, as the full QS network that regulates both bioluminescence and host colonization receives input from many environmental factors [22,40]. However we can still ask which properties of the *V.fischeri *LuxI/LuxR system could make an antagonistic interaction between 3OC6HSL and C8HSL advantageous. We used our experimental and modeling results to quantify the signal-transmission property of the two-HSL system. We calculated the mutual information between *lux *output and the signal inputs [23-25] by modeling the population-averaged *lux *activation *F*(*X, Y*) with the competitive inhibition model (where *X *and *Y *are scaled variables corresponding to the relative saturation of LuxR by 3OC6HSL and C8HSL respectively), and then modeling the noise in *F *by a gamma distribution that captures the coefficient of variation observed in our single-cell experiments (Figure 7 and *Methods*). In the absence of *any *other information about signal inputs -* i.e*. using the simplest assumption that all input combinations (*X, Y*) are equally likely *a priori *- the calculation leads to a surprisingly low estimate for the mutual information, *I*(*Z*,(*X, Y*)) ≈ 0.53 bits. Even with its two signal inputs, the output *F*(*X, Y*) of the LuxI/LuxR system transmits less information about its inputs than would a simple ON/OFF switch. By contrast, Mehta *et al*. estimated ~1.2-1.7 bits of mutual information between the output and two inputs (AI1 and AI2) of the phosphorelay system in *V.harveyi *QS. The noisy performance (CV ~ 0.5) and gradual switching of LuxI/R significantly degrades its sensing capability, in comparison to the *V.harveyi *circuit.

Therefore we find no indication that the second (C8HSL) autoinducer enhances the precision of signal response in the *V.fischeri *LuxI/R system. However the poor information throughput of this system does suggest a different perspective on the idea [13] that C8HSL conserves energy resources by delaying induction of *lux *at low population densities. At the population-average level, the same delay in activation could be achieved by raising the activation threshold for the 3OC6HSL signal. However at the single cell level, the presence of any 3OC6HSL induces a highly heterogeneous response, with some cells luminescing much more brightly than average. Thus, even if the threshold is set very high, a few cells will waste energy by emitting light during early growth. One benefit of producing a small concentration of C8HSL is that it collapses the bioluminescence distribution, suppressing the most active emitters and conserving metabolic energy. Simultaneous synthesis of C8HSL and 3OC6HSL may therefore reduce luminescent output by virtually all cells, at least until 3OC6HSL attains high concentration. In this sense the crosstalk from the C8HSL signal does not improve the environment-sensing precision of LuxI/R at steady state, but it may tend to compensate for the noisy performance of the LuxI/R switch by suppressing the switch for as long as possible during growth and colonization. It would be intriguing to see if dynamical models that accurately capture the noise in the circuit and the temporal accumulation of HSL can characterize this behavior quantitatively.

## Conclusions

Although multiple-input quorum-sensing systems are widespread in the microbial world, the mechanisms by which they combine and process information from parallel signal inputs are in general poorly understood. One of the intriguing properties of the *V.fischeri *QS network is that it employs two autoinducer signals that can act competitively or antagonistically in regulating the *lux *genes. In order to understand the possible advantages of this competitive interaction we have studied the response of individual *V.fischeri *to combinations of HSL signals. The population-averaged, steady state activation of *lux *by the two HSL signals is readily described by a quantitative, competitive inhibition model. Our measurements of *lux *activation in individual cells show a noisy response, with the LuxI/R circuit conveying less than one bit of mutual information between its HSL signal inputs and its *lux *output. Further the data provide no indication that either the dynamics of the *lux *response or the heterogeneity in that response are sensitive to different combinations of signals that generate the same population-averaged output. In this sense the second HSL signal input appears to provide little if any additional information to the *lux *system. These findings may instead suggest a dynamical role in which the production of C8HSL signal provides an energetic advantage by suppressing sensitivity of the luminescence switch during the growth of a population.

## Methods

### Fluorescence and luminescence response of bulk culture

*V.fischeri *mutant JB10 is a derivative of the ES114 strain in which a chromosomal *gfp *reporter is inserted into the *lux *operon by allele exchange, producing *luxI-gfp-luxCDABEG *[40]. We prepared JB10 from a glycerol stock and grew the cells to exponential phase in defined artificial seawater medium [41] to which was added 0.3% casamino acids. Cells were then diluted and regrown to OD ~ 0.1-0.3 in fresh medium, washed three times, and then rediluted 100× into a 96-well assay plate containing fresh medium. The individual wells were preloaded with an 11 × 8 array of concentrations of the two HSL autoinducers *N*-3-oxohexanoyl-*L*-homoserine lactone (3OC6HSL, Sigma #K3007) and *N-*octanoyl-*L*-homoserine lactone (C8HSL, Cayman Chemical Co. #10011199). The well plate was then incubated in a Biotek Synergy 2 plate reader at 25°C, giving a growth rate 1.1 ± 0.1 hr^-1^. Optical density was measured at 600 nm, and GFP fluorescence was measured using a 485/20 nm excitation filter and a 528/20 nm emission filter. The optical density, luminescence and GFP fluorescence values for each well were recorded at regular intervals during exponential growth (Figure 2). Data collected early in growth (*t *< 12 hrs) showed a sensitive dependence on the exogenous levels of both HSLs, indicating that endogenous HSL did not accumulate significantly during this interval.

### Competitive inhibition model for bulk response

In order to generate a mathematical representation of the *lux *response, as a function of the 3OC6HSL and C8HSL signals, we fit the JB10 well-plate data (fluorescence *vs *HSL concentrations) to the competitive inhibition model of Figure 1[11,13]. In this model *lux *is regulated primarily through competition between C8HSL and 3OC6HSL to form LuxR complexes that act as transcriptional activators for the *lux *genes. The action of C8HSL on LuxR synthesis through AinR and the phosphorelay is not considered. We assume that 3OC6HSL and C8HSL diffuse freely across the cell envelope and form multimeric complexes with LuxR. We allow an arbitrary degree of multimerization but we do not consider heterocomplexes (*i.e*. involving both C8HSL and 3OC6HSL). Although it is simple to include the weak activation of *lux *by C8HSL-LuxR, which is evident in the bioluminescence data at low 3OC6HSL concentrations, this activation is scarcely visible in the GFP fluorescence data that is the target of our modeling. Therefore we omitted this mechanism from our model and considered C8HSL only in its role as a competitor for LuxR. That is, we assume that the GFP fluorescence is proportional to the concentration of the 3OC6HSL-LuxR complex. More biochemical accuracy could be included by introducing extra parameters, but the simpler model appears sufficient to describe the JB10 data.

The model allows C8HSL and 3OC6HSL to form multimeric complexes (of degree *m *and *n *respectively) with LuxR,

$$\begin{array}{c} m\;C8HSL+m\;LuxR\Leftrightarrow {\left(C8HSL-LuxR\right)}_{m}\\ n\;3OC6HSL+n\;LuxR\Leftrightarrow {\left(3OC6HSL-LuxR\right)}_{n} \end{array}$$

where the Hill coefficients *n *and *m *are not assumed to be integers. These equilibria are characterized by two dissociation constants, *K*_1 _and *K*_2_:

$$\begin{array}{c} K_{1}^{2m-1}=\frac{{\left[LuxR\right]}^{m}{\left[C8HSL\right]}^{m}}{\left[{\left(C8HSL-LuxR\right)}_{m}\right]}\\ K_{2}^{2n-1}=\frac{{\left[LuxR\right]}^{n}{\left[3OC6HSL\right]}^{n}}{\left[{\left(3OC6HSL-LuxR\right)}_{n}\right]} \end{array}$$

*K_1 _*and *K_2 _*are defined so as to have units of concentration, regardless of the values of *m *and *n*. If [LuxR_0_] is the average total concentration of LuxR, including complexes, then

(3) $$\left[\text{Lux}{\text{R}}_{0}\right]=\left[\text{LuxR}\right]+n\left[{\left(3\text{OC}6\text{HSL}-\text{LuxR}\right)}_{\text{n}}\right]+m\left[{\left(\text{C}8\text{HSL}-\text{LuxR}\right)}_{\text{m}}\right]$$

As we do not measure the actual LuxR copy number (although see [42]), it is convenient to redefine the dissociation constants in terms of [LuxR_0_] and a dimensionless concentration *r*:

(4) $$\begin{array}{l} k_{1}^{m}=\frac{r^{m}{\left[C8HSL\right]}^{m}}{\left[{\left(C8HSL-LuxR\right)}_{m}\right]/\left[LuxR_{0}\right]}\\ k_{2}^{n}=\frac{r^{n}{\left[3OC6HSL\right]}^{n}}{\left[{\left(3OC6HSL-LuxR\right)}_{n}\right]/\left[LuxR_{0}\right]} \end{array}$$

Here *k_1 _*and *k_2 _*have dimensions of (autoinducer) concentration. Then Eqn. (3) becomes

(5) $$1=r+mr^{m}\frac{{\left[C8HSL\right]}^{m}}{k_{1}^{m}}+nr^{n}\frac{{\left[3OC6HSL\right]}^{n}}{k_{2}^{n}}$$

Starting from the HSL concentrations and an initial guess for the parameters (*k_1_, k_2_, m, n*), we solve Eqn. (5) to find *r*. Then Eqn. (4) gives the concentrations (relative to LuxR_0_) of the two multimer species. We compare the model to the well-plate data by assuming that the GFP fluorescence *F *is a linear, non-saturating function of the two multimer concentrations:

(6) $$\begin{array}{c} F=F_{0}+a_{1}\frac{\left[{\left(C8HSL-LuxR\right)}_{m}\right]}{\left[LuxR_{0}\right]}+\ldots \\ \;a_{2}\frac{\left[{\left(3OC6HSL-LuxR\right)}_{n}\right]}{\left[LuxR_{0}\right]} \end{array}$$

Here *F*_0_, *a*_1 _and *a*_2 _are positive constants (see *Results*). As explained above, *a*_1 _is evident in luminescence but is scarcely detectable in the fluorescence; setting *a*_1 _= 0 does not impair the fit. Then the shape of the *2D *surface *F*(3OC6HSL, C8HSL) is determined solely by the four parameters *k_1_, k_2_*, *n*, and *m*, while the parameters *F_0 _*and *a_2 _*provide an instrument-dependent offset and amplitude that scale the *2d *model *F *surface onto the measured values. We estimate the four model parameters through a nonlinear least squares fit of the fluorescence response surfaces *F*(3OC6HSL, C8HSL) measured at optical densities 0.05-0.15 cm^-1 ^to Eqn. (6), with the scale parameters *a*_2 _and *F*_0 _determined by linear regression. This provides a parametrization of the average response *F *as a function of the two HSL inputs (Table 1).

The data do not require that *m *and *n *are different. As Table 1 indicates, the fit yields similar values for the two Hill coefficients (*m *= 1.1 ± 0.4 and *n *= 1.35 ± 0.05), and in fact we obtain a very similar fit if we assume that the same coefficient applies for both autoinducers (*m *= *n *= 1.2 ± 0.2). Table 1 also shows (as expected from Eqn. (1)) that we obtain similar parameters when we fit Eqn. (6) to the square root of the measured luminescence *L*^1/2 ^rather than to the GFP fluorescence *F*.

### Microfluidic studies of individual cells

To measure the effect of exogenous HSL signals on *lux *expression in individual JB10 cells we loaded cells into microfluidic perfusion chambers that supplied a flow of medium containing exogenous 3OC6HSL and C8HSL. Each microfluidic device consisted of three parallel and unconnected channels (Figure 4), with each channel having width 400 μm (parallel to the observation window but perpendicular to the fluid flow), depth 10-15 μm (perpendicular to the observation window), and length (parallel to observation window and to fluid flow) 10 mm. The devices were fabricated from PDMS silicone elastomer (Sylgard 184, Dow-Corning Corporation) by a standard soft-lithographic method in which a PDMS replica is cast from a reactive ion-etched silicon master [43]. The device channels were sealed by a glass coverslip bonded to the PDMS. In order to promote cell adhesion to the interior of the glass window, we coated the interior of the device by filling it with a solution of poly-*L*-lysine (1 mg/ml, MW 300 000) and incubating it for 24 hours at 5°C, prior to cell injection. This provided stable adhesion of the *V.fischeri *to the glass window.

JB10 cells for microfluidic studies were prepared in exponential phase as for the 96-well assay above: We grew cells to exponential phase in defined artificial seawater medium with casamino acids [41], then washed (3×) and rediluted the cells, and then regrew them to OD (600 nm) = 0.015-0.03 cm^-1 ^in fresh medium. Once the cells and the microfluidic device were prepared, we flushed the poly-*L*-lysine solution by pumping the JB10 culture into all three parallel channels at 1-2 ml/hr with a syringe pump. We then placed the device (with glass window facing downward) on the stage of a Nikon TE2000U microscope and reduced the flow rate to ~0.02 ml/hr. At this slow flowrate the cells gradually settle and adhere to the glass window. Once a sufficient number of cells had adhered to the window (requiring 15-30 minutes), we supplied autoinducer by connecting the device inputs to syringe pumps that delivered defined medium containing exogenous 3OC6 HSL and/or C8HSL. Each of the three channels was supplied with a different combination of HSLs, flowing at a rate ~0.02 ml/hr during fluorescence measurements.

The 0.02 ml/hr flow rate of medium corresponds to an average flow velocity of ~1 mm/s within each channel. Both the device design and experimental testing ensured that this flow was sufficiently uniform and rapid to wash away endogenous (natively produced) autoinducer that might otherwise affect activation of the *lux *genes. First of all, control experiments in our flow system showed that - in the absence of any exogenous autoinducer (HSL) - *gfp *expression from the *lux *reporter strain was at its baseline level (and luminescence was unobservable). Moreover, the physical parameters of the flow system make it highly implausible that spatial heterogeneity in the flow could develop or allow experimentally relevant concentrations of HSL to accumulate near any of the cells under observation: First, the dimensions of the device and the flow rate of growth medium lead to fluid flow at a very low Reynolds number (*Re *~0.03). At this *Re *the flow velocity profile is highly uniform across the width and length of the flow chamber, up to within ~10 μm of the chamber edges [44]. Second, the only significant heterogeneity in this flow velocity profile occurs along the depth of the channel (*i.e*. perpendicular to the window), which is 10-15 μm. However HSL requires only ~0.1 s to diffuse this distance. This is so much faster than other relevant time scales in the experiment that a meaningful HSL gradient cannot be established in this direction. Third, the chamber volume and the 1 mm/s flow rate together indicate that the entire volume of the cell chamber region (10 mm length) is completely flushed every ~10 seconds. However the only cells occupying the chamber (and producing HSL) are those forming a sparse single layer (cells are typically spaced > 20 μm apart) on the chamber window. Literature estimates of HSL production rates in *V.fischeri *indicate that such a sparse layer of individual cells, within a chamber that is flushed at this rate, would not be able to generate an endogenous HSL concentration above ~100 pM [20]. This concentration is at least two orders of magnitude smaller than the exogenous HSL concentrations that we are providing.

Finally, if the cells did generate enough HSL to affect local concentrations, we would expect that cells downstream would in general express more GFP than cells upstream. More generally we would expect the correlation *C_ij _*between the GFP fluorescence *F_i_, F_j _*of a pair of cells *i, j*

(7) $$C_{ij}=\left(F_{i}-\mu \right)\left(F_{j}-\mu \right)∕\sigma^{2}$$

to depend on their spatial separations *x_ij_*, *y_ij_*, or *r_ij_*. (Here μ is the mean cell fluorescence and σ^2 ^is the variance in *F*.) We analyzed our data for such spatial correlations and found none. For example, Figure 4 shows no relationship between *C_ij _*and *r_ij_*: the *gfp *expression of two neighboring cells is no more similar than that of two distant cells. In short the data and the system design argue strongly against any autoactivation of (or local crosstalk between) the individual cells under observation.

### Characterizing heterogeneity in *lux *activation

The three-channel device allowed us to collect the fluorescence histogram of cells under three different HSL signal combinations, as it evolved over 4-5 hours. Once HSLs were introduced to the device at *t = *0, we collected phase contrast and fluorescence image pairs for each channel (HSL combination) at intervals of 20 minutes, using a 20×/0.50 NA phase objective and a GFP filter cube. Images were recorded by a Coolsnap HQ2 camera (Photometrics) at -30°C and corrected in software for dark current and flat-field.

For each experimental condition we evaluated the fluorescent emission from (typically) ~200 individual cells by first determining the physical locations (pixel coordinates) of single cells in a phase contrast image. We then used a homemade Matlab code to evaluate the fluorescence per cell pixel in the associated fluorescence image by summing the fluorescence emission (relative to background) of the contiguous bright pixels associated with the cell's pixel coordinates. Normalizing the histogram of individual cell fluorescence values gives a distribution *P*(*F *| ([3OC6HSL],[C8HSL])), representing the probability of cell fluorescence *F *given the HSL input concentrations.

### Calculating the mutual information

By combining mathematical parametrizations of both the *lux *response, *F*([3OC6HSL],[C8HSL]) and the probability distribution *P*(*F *| ([3OC6HSL],[C8HSL])), we calculate the mutual information [23] between the HSL signal inputs and *lux *output. The signal concentrations are inconvenient parameters for this calculation because only an infinite concentration of autoinducer can saturate the response. In their analysis of *V.harveyi *QS, Mehta *et al*. [25] defined new coordinates that describe the state of saturation of the autoinducer receptors. In similar fashion we replace [3OC6HSL] and [C8HSL] with coordinates *X *and *Y *that describe the state of the association equilibria for the HSL-LuxR complexes:

(8) $$\begin{array}{c} X=\frac{{\left[3OC6HSL\right]}^{n}}{{\left[30C6HSL\right]}^{n}+k_{2}^{n}}\\ Y=\frac{{\left[C8HSL\right]}^{m}}{{\left[C8HSL\right]}^{m}+k_{1}^{m}} \end{array}$$

*X *= 1 or *Y *= 1 corresponds to complete saturation of the 3OC6HSL-LuxR or C8HSL-LuxR binding equilibrium respectively. With these coordinates the response surface *F*(*X,Y*) for the competitive inhibition model has a simple shape (Figure 7) that is independent of the parameters *k_1_*, *n*, *k_2_*, *m*.

The mutual information between a combination of inputs (*X,Y*) and the output *F *is then calculated as:

(9) $$\begin{array}{c} I\left(F;\left(X,Y\right)\right)=\int dFdXdY\;P\left(F,\left(X,Y\right)\right)\;\log_{2}\;\frac{P\left(F,\left(X,Y\right)\right)}{P\left(F\right)P\left(X,Y\right)}\\ =\int dFdXdY\;P\left(F|\left(X,Y\right)\right)\;P\left(X,Y\right)\log_{2}\frac{P\left(F|\left(X,Y\right)\right)}{P\left(F\right)} \end{array}$$

Here *P*(*F*) is the probability of finding output *F*, in the absence of any knowledge of the input (*X, Y*). *P*(*F*|(*X, Y*)) is the probability of *F*, given the combination (*X, Y*). *P*(*F*,(*X, Y*)) is the probability of observing the particular combination *F*, (*X, Y*):

$$P\left(F,\left(X,Y\right)\right)=P\left(F\;|\left(X,Y\right)\right)P\left(X,Y\right)$$

These probability distributions are normalized as follows:

$$\begin{array}{c} P\left(F\right)=\int dXdY\;P\left(F,\left(X,Y\right)\right)=\cdots \\ =\int dXdY\;P\left(F|\left(X,Y\right)\right)P\left(X,Y\right)\\ \int dF\;P\left(F\right)=1\\ \int dF\;P\left(F|\left(X,Y\right)\right)=1\\ \int dFdXdY\;P\left(F,\left(X,Y\right)\right)=1\\ \int dXdY\;P\left(X,Y\right)=1 \end{array}$$

To evaluate Eqn. (9) we model *P*(*F*,(*X, Y*)) as the gamma distribution that has the same mean and variance as observed in the bulk and single-cell measurements respectively. The calculation also requires an estimate of *P*(*X, Y*), the prior probability of a particular combination (*X, Y*). *P*(*X, Y*) is not so easily predicted. However, given that *X *and *Y *are both bounded by 0 and 1 we made the straightforward assumption that *P*(*X, Y*) = *constant*. The mutual information Eqn. (9) is then found to be *I ≈ *0.53 bits. However this result is not sensitive to our assumptions about the prior probability: various *P*(*X, Y*) functions that were strongly bimodal in both *X *and *Y*, and either symmetric or asymmetric in *X *vs. *Y *[25], all gave similar values of *I *≈ 0.5 bits.

## List of Abbreviations

C8HSL: C8 homoserine lactone, *N-*octanoyl-*L*-homoserine lactone; GFP: green fluorescent protein; HSL: homoserine lactone; JB10: *Vibrio fischeri *strain JB10; QS: quorum sensing; 3OC6HSL: 3-oxo-C6 homoserine lactone, *N*-3-oxohexanoyl homoserine lactone.

## Authors' contributions

PDP-Acquired and analyzed data; JTW-Acquired data; SJH-Designed research, analyzed data, and drafted the manuscript. All authors read and approved the final manuscript.
